# Dr. Geo. O. Williams

**Published:** 1916-05

**Authors:** 


### OBITUARY

**Readers are requested to report promptly the death of all physicians in Western New York, or former residents of this region, or graduates of any medical school in Western New York, and to notify the families of the deceased of our desire to publish adequate Obituary notices.**

**Dr. Geo. O. Williams,** Albany 1866, died at his home in Greene, Feb. 24, of bronchopneumonia, aged 72.

